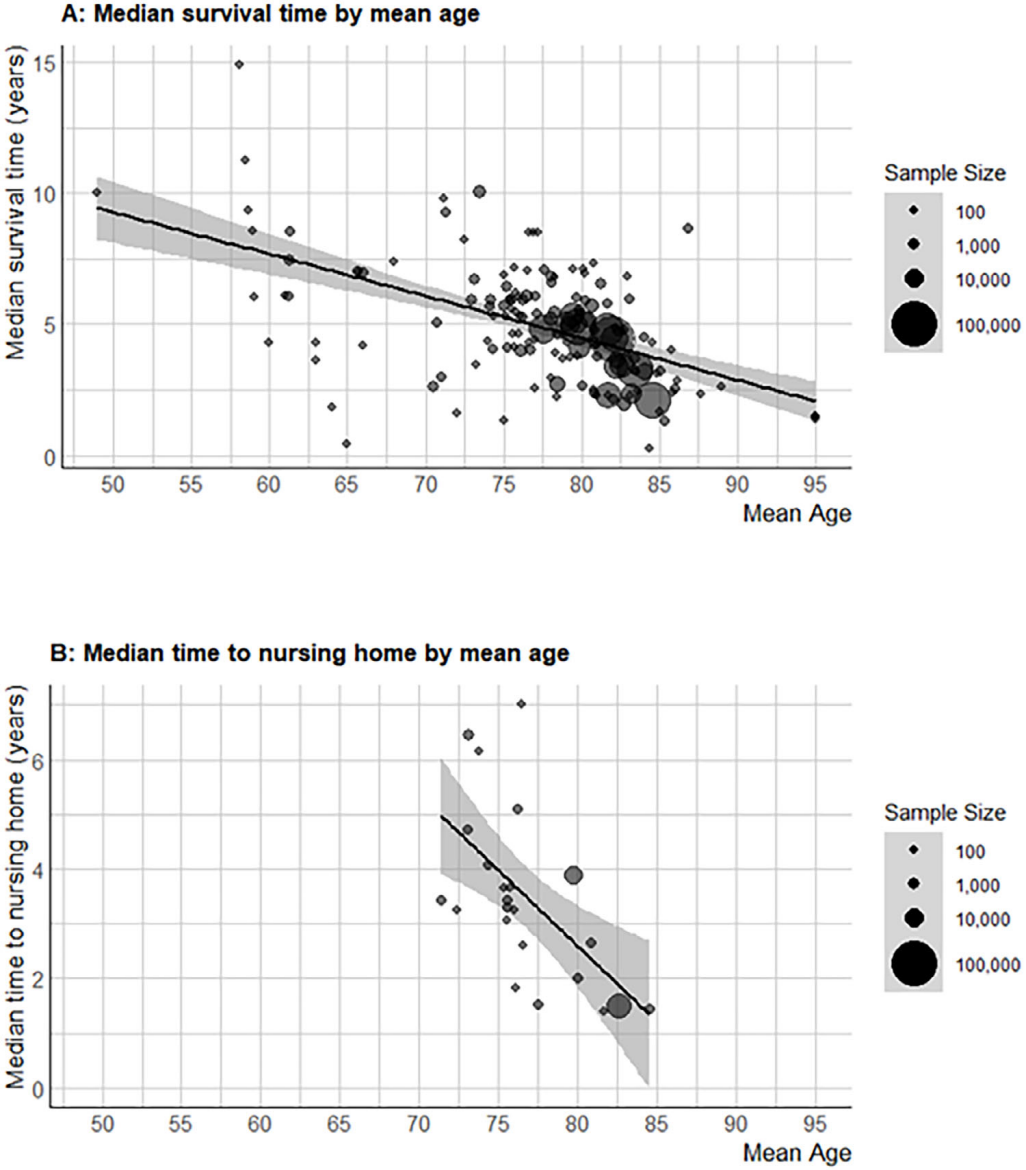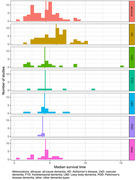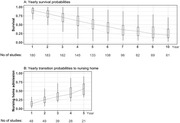# Survival and time till nursing home admission in persons with dementia: a systematic review and meta‐analysis

**DOI:** 10.1002/alz.087319

**Published:** 2025-01-09

**Authors:** Chiara C. Brück, Sanne S. Mooldijk, Lieke M. Kuiper, Frank J. Wolters

**Affiliations:** ^1^ Department of Public Health, Erasmus MC University Medical Center, Rotterdam Netherlands; ^2^ Department of Epidemiology, Erasmus MC University Medical Center, Rotterdam Netherlands; ^3^ Department of Internal Medicine, Erasmus MC University Medical Center, Rotterdam Netherlands; ^4^ Department of Epidemiology, Erasmus University Medical Center, Rotterdam Netherlands; ^5^ Department of Radiology & Nuclear Medicine and Alzheimer Center, Erasmus MC, Rotterdam Netherlands

## Abstract

**Background:**

Accurate prognosis after a dementia diagnosis is important for patient information and advanced care planning, but available information is fragmented, with inconsistent estimates that are often restricted to survival. We aimed to summarise the literature to assess survival times and time to nursing home after dementia diagnosis and determine the impact of clinical characteristics and study design.

**Method:**

We systematically searched MEDLINE and EMBASE for studies reporting time from dementia diagnosis to nursing home admission or death. Two reviewers independently extracted information on study design, patient population, disease characteristics, and outcomes, including semi‐automated quantification of Kaplan‐Meier survival curves. We used a modified Newcastle‐Ottawa Scale for study appraisal and performed meta‐regression analyses to identify determinants of prognosis.

**Result:**

Of 212 included studies, 188 reported on survival (1,255,916 participants) and 70 on nursing home admission (229,925 participants). Overall, median survival was 4.7 years (interquartile range: 3.4‐6.0), and this differed substantially with patient demographics and disease characteristics. Survival varied with age and was on average 6.6 years among studies with a median age of 65 years, and 3.7 years with a median age of 85 (Figure 1A). Shorter observed survival among women than men was explained entirely by later disease onset in women. Survival was longer among patients with Alzheimer’s disease compared to all‐cause dementia (Figure 2), and longer in more recent studies (1.3 years comparing studies ≥2010 to <1990). Patients in Asia lived longer after diagnosis than their counterparts in the U.S. and Europe (1.1‐ and 1.3‐years difference, respectively). Survival probabilities were 88%, 69%, 52% and 26% after 1, 3, 5 and 10 years of follow‐up, respectively (Figure 3A). Taken together, variation in methodology and clinical characteristics explained 50% of heterogeneity in survival among studies. Median time to nursing home admission was 3.3 years (IQR 2.2‐4.0; Figure 1B), with 15% of patients being admitted after 1 year, increasing to 36% at 3, and 57% at 5 years from diagnosis (Figure 3B), which was influenced only by age.

**Conclusion:**

Patients with dementia have an average life‐expectancy of five years at time of diagnosis, of which two years are lived in nursing homes.